# ‘Energy-Dense, High-SFA and Low-Fiber’ Dietary Pattern Lowered Adiponectin but Not Leptin Concentration of Breast Cancer Survivors

**DOI:** 10.3390/nu13103339

**Published:** 2021-09-24

**Authors:** Mohd Razif Shahril, Nor Syamimi Zakarai, Geeta Appannah, Ali Nurnazahiah, Hamid Jan Jan Mohamed, Aryati Ahmad, Pei Lin Lua, Michael Fenech

**Affiliations:** 1 Centre for Healthy Ageing and Wellness (H-CARE), Faculty of Health Sciences, Universiti Kebangsaan Malaysia, Jalan Raja Muda Abdul Aziz, Kuala Lumpur 50300, Selangor, Malaysia; mf.ghf@outlook.com; 2 School of Nutrition and Dietetics, Faculty of Health Sciences, University Sultan Zainal Abidin, Gong Badak Campus, Kuala Nerus 21300, Terengganu, Malaysia; norsyamimizakarai@gmail.com (N.S.Z.); nurnazahiahali@unisza.edu.my (A.N.); aryatiahmad@unisza.edu.my (A.A.); 3 Department of Nutrition, Faculty of Medicine and Health Sciences, Universiti Putra Malaysia, Seri Kembangan 43400, Selangor, Malaysia; geeta@upm.edu.my; 4 Nutrition and Dietetics Programme, School of Health Sciences, Universiti Sains Malaysia, Kubang Kerian 16150, Kelantan, Malaysia; hamidjan@usm.my; 5 Faculty of Pharmacy, Universiti Sultan Zainal Abidin, Besut Campus, Besut 22200, Terengganu, Malaysia; peilinlua@unisza.edu.my; 6 Health and Biomedical Innovation, UniSA Clinical and Health Sciences, University of South Australia, Adelaide, SA 5000, Australia

**Keywords:** HMW adiponectin, leptin, dietary patterns, breast cancer survivors

## Abstract

Dietary pattern (DP) and its relationship with disease biomarkers have received recognition in nutritional epidemiology investigations. However, DP relationships with adipokines (i.e., adiponectin and leptin) among breast cancer survivors remain unclear. Therefore, we assessed relationships between DP and high-molecular weight (HMW) adiponectin and leptin concentration among breast cancer survivors. This cross-sectional study involved 128 breast cancer survivors who attended the oncology outpatient clinic at two main government hospitals in the East Coast of Peninsular Malaysia. The serum concentration of HMW adiponectin and leptin were measured using enzyme-linked immunosorbent assay (ELISA) kits. A reduced rank regression method was used to analyze DP. Relationships between DP with HMW adiponectin and leptin were examined using regression models. The findings show that with every 1-unit increase in the ‘energy-dense, high-SFA, low-fiber’ DP z-score, there was a reduction by 0.41 μg/mL in HMW adiponectin which was independent of age, BMI, education level, occupation status, cancer stage, and duration since diagnosis. A similar relationship with leptin concentration was not observed. In conclusion, the ‘energy-dense, high-saturated fat and low-fiber’ DP, which is characterized by high intake levels of sugar-sweetened drinks and fat-based spreads but low intake of fruits and vegetables, is an unhealthy dietary pattern and unfavorable for HMW adiponectin concentration, but not for leptin. These findings could serve as a basis in developing specific preventive strategies that are tailored to the growing population of breast cancer survivors.

## 1. Introduction

With the increase in the number and life expectancy of breast cancer survivors, the focus of cancer care has shifted towards better survivor care, including nutritional interventions and lifestyle changes. Dietary pattern has received considerable critical attention in nutritional epidemiology as one of the potential factors in modifying cancer risk, recurrence, or mortality [1,2]. Sotos-Prieto et al. [3] found that the risk of all-cause and cause-specific mortality was lowered with better diet quality, conceivably due to the beneficial effects of improvements in dietary intake such as increasing intake of whole grains, vegetables, fruits, and fish or n-3 fatty acids. In addition, other studies also suggested that many cancer survivors were interested in shifting their diet towards healthy diet practice after been diagnosed with cancer [4,5,6,7].

In current nutrition epidemiologic studies, because diet is a complex entity with many interactions between foods, interest in the exploration of dietary pattern (DP) analysis has been increasing [8,9,10]. The determination of DP was described previously as an examination of the totality of the diet which provides a more holistic description of actual dietary exposures [11]. A healthy DP consists of a high intake of legumes, fruits and vegetables, while limiting energy-dense foods and sodium is more important for the prevention of chronic diseases instead of the intake or exclusion of specific food items or nutrients [12]. Therefore, in this context, DP analysis which focuses on a combination of several foods can provide more detailed information about diet and disease risk.

Additionally, in cancer research, the most abundant adipokines, i.e., adiponectin and leptin, are gaining recognition as modifiable risk factors due to their links with obesity and obesity-related cancer [13]. It has been shown that obesity is associated with a 30% greater risk of mortality in all types of breast cancer, while being physically active has been associated with a 30% lower risk [14]. Adiponectin, a peptide with 244 amino acids, has a strong reverse correlation with adiposity and is recognized as anti-inflammatory [13]. In contrast, leptin, a 16 kDa protein, is a product of the obesity gene (Ob/Ob) which increases in concentration with adiposity and has a direct mitogenic action on breast cancer cells or acts indirectly to promote the production of estrogen-receptor and resistance to insulin [15,16].

Several modifiable behaviors, such as diet and exercise, that can be effective in preventing obesity and beneficially altering circulating adipokine levels may be critical both for cancer prevention and for improved health outcomes after a diagnosis of breast cancer [17,18]. Therefore, it is crucial to discover the types of DP which are characterized by the inclusion of dietary factors that have been hypothesized to be associated with breast cancer survival and with altering adipokine concentration. Reviews on the relationship between DP with adiponectin and leptin [8] suggested that a healthy DP such as a diet high in vegetables, fruits and lean meat was negatively associated with serum leptin concentration [17,19] but positively related with adiponectin concentration [20,21,22].

Nevertheless, many previous studies have not only focused on a single nutrient or specific foods, but also did not use reduced rank regression (RRR) method in determining DP of breast cancer survivors although RRR was reported as a useful method for examining the role of diet concerning health outcome or disease risk [23]. In addition, the relationships between breast cancer survivors’ DP and their serum adipokines concentration are also not well established. Therefore, this present study was conducted to identify the DP with selected dietary factors associated with breast cancer survivorship in the East Coast of Peninsular Malaysia by determining its relationship with serum adipokine (HMW adiponectin and leptin) concentrations using the reduced rank regression method.

## 2. Materials and Methods

### 2.1. Study Design and Sample

This cross-sectional study was conducted among 128 breast cancer survivors who had completed the main treatment modalities (surgery, chemotherapy and/or radiotherapy) in the past six months or more. The respondents were recruited from two main government hospitals, i.e., Hospital Raja Perempuan Zainab II in Kota Bharu, Kelantan and Hospital Sultanah Nur Zahirah in Kuala Terengganu, Terengganu based on purposive sampling. The ethical approval for this present study was obtained from the Medical Research and Ethics Committee of the Ministry of Health Malaysia (NMRR-14-1618-23717). Breast cancer survivors who were pregnant, had secondary or recurrent cancer, stage IV cancer, had cardiovascular disease, orthopedic problems or any other medical conditions were excluded from the study. Only survivors who provided informed consent were included as respondents in this study.

### 2.2. Dietary Assessments

An interviewer-administered semi-quantitative food frequency questionnaire (FFQ) was used to assess dietary information of breast cancer survivors. The FFQ was modified from the Malaysia Adult Nutrition Study [24] and validated against a weighed food record [25]. This FFQ included 195 food and beverage items commonly consumed among Malaysian adults. Respondents were asked to specify their consumption frequency of each food item on a daily, weekly, monthly, or yearly basis. Common household measures including cups, spoons, bowls, plates as well as the amount of food in the form of fractions such as one whole, half one whole, a piece or one slice were used to better estimate the actual portion size. Subsequently, portion sizes consumed from each food item were converted to daily intake in grams by multiplying the frequency of consumption with exchange factors as described in a previous study [26]. Energy and nutrients contents of foods were calculated using a database which was developed based on the Nutrient Composition of Malaysian Food [27] and the United States Department of Agriculture (USDA) food composition database [28], as described elsewhere [29].

### 2.3. Dietary Pattern Analysis

Dietary pattern was determined using RRR, a statistical technique designed by combining the advantages of the exploratory and hypothesis-oriented approaches to dietary patterns. In brief, the RRR method uses two different sets of variables, i.e., a set of independent variables or predictors, generally dietary components, and a set of response variables, selected based on the a priori hypothesis that they are related to the outcome of interest. Macronutrients and dietary biomarkers associated with the disease of interest are often used as response variables. Therefore, in the present study, the RRR model included three dietary factors, i.e., dietary energy density (DED), saturated fat (SFA) and dietary fiber (DF). These dietary factors were selected based on evidence derived from the World Cancer Research Fund/American Institute for Cancer Research (WCRF/AICR) guidelines for breast cancer prevention and recurrence [30]. These guidelines imply that women who eat more food containing fiber, both before and after diagnosis, may have a lower risk of dying from breast cancer. Conversely, a diet high in fat, especially saturated fat, before and after diagnosis may have an increased risk of dying after a breast cancer diagnosis.

In the same vein, relevant studies supported the hypothesis that fat intake, SFA and plant fiber are associated with breast cancer risks and mortality [12,31,32]. For dietary energy density (DED), innumerable studies have observed the association between DED and weight status, including studies evaluating the relationship between DED and markers for metabolic syndrome [33,34], and a positive relationship was found between DED with BMI [35,36]. Weight status is also a known major risk factor for cancer generally, including breast cancer [37,38].

Dietary energy density was calculated by dividing total food energy intake (kcal) by total food weight (g), but excluded all beverages because of their disproportionate influence on total dietary energy density value. This method for deriving DED was also recommended by Livingstone and McNaughton [39]. In order to report adjusted intake for energy consumption, SFA and dietary fiber intake values were expressed as a percentage of energy contribution and grams per kcal, respectively.

A total of 31 food groups (g/d) which were grouped according to their nutrient profiles and food group categories, such as whole grains, refined grains, green leafy vegetables, cruciferous vegetables, bean-based vegetables, fruits, sweet dessert, processed meat group, etc., were used as predictors in this RRR analysis. Three factors were identified for DP analysis in this study. Only factors that explained the most variation in all response variables were chosen to be further investigated with serum adipokines concentration in the present study. Each of the breast cancer survivors received a z-score for the DP identified, discriminating how strongly their dietary intakes linked with the DP.

### 2.4. Adipokines

Approximately, five milliliters of fasting blood from breast cancer survivors were drawn by venipuncture and transferred in a red-top tube, a BD Vacutainer^®^ Plus Plastic Serum Tubes containing no anticoagulant, during the data collection. The tube was centrifuged at 3500 rpm, for 10 min at 4 degrees Celsius. Serum was transferred into the 1.5 L tube and stored at −80 degrees Celsius. The present study measured two target proteins: HMW adiponectin and leptin by using the enzyme-linked immunosorbent assay (ELISA) method following a typical two-step capture or ‘sandwich’ type assay for the detection of the target protein. The ELISA kits used were Human Adiponectin Immunoassay Kit Cat.No.47-ADPHU-E01 and Human Leptin Immunoassay Kit Cat.No.11-LEPHU-E01 (American Laboratory Products Company (ALPCO) Diagnostics, Salem, NH, USA).

### 2.5. Covariates Assessment

#### 2.5.1. Socio-Demographic and Clinical Characteristics

The present study used a set of questionnaires consisting of sociodemographic and clinical characteristics and was interviewer-administered on a one-to-one basis. Socio-demographic questions consisted of age, home address, monthly income, ethnicity, marital status, and education level, as well as job status. Clinical characteristics questionnaires included the year cancer was diagnosed, stage of cancer, treatments and medications, other health problems faced by the respondents as well as complications experienced by the respondents after their cancer’s treatment.

#### 2.5.2. Anthropometric and Body Compositions Assessments

Anthropometric and body composition assessments such as body weight and per centage of body fat were measured using a body composition analyzer (Tanita BC-587, TANITA Corporation, Tokyo, Japan). Height was measured to the nearest 0.1 cm by using a mobile stadiometer (Seca 217, Seca, Hamburg, Germany) and waist measurement was taken by using a measuring tape (Seca 201, Seca, Hamburg, Germany) at the smallest waist area.

### 2.6. Statistical Analysis

All data were analyzed using IBM SPSS Statistics for Windows Version 22.0 software (IBM Corporation, Armonk, NY, USA), except for the dietary pattern, for which the partial least squares procedure with reduced rank regression option was used, analyzed using SAS Software Version 9.4 (SAS Institute, Cary, NC, USA). Descriptive statistics including the mean, standard deviation and range were used to present the respondent’s serum adipokines concentration. Simple linear regression was performed to identify the possible independent factors related to adipokines concentration without considering any confounder. Next, multivariate regression analysis was conducted to analyze the relationship between the mean DP z-scores of breast cancer survivors and their serum adipokines (HMW adiponectin and leptin) concentrations including the adjusted variables which could be biologically important during model development were included. Overall, age, BMI, cancer stage, duration since diagnosis, education level and occupation were the selected confounders for the link between DP and adipokines concentration in this study.

## 3. Results

Table 1 describes the characteristics of breast cancer survivors, including the socio-demographic, anthropometric measurement and adipokine (HMW adiponectin and leptin) profile. In summary, the majority of the respondents were Malay (94.5%), married (77.3%) and had secondary education (59.4%). The majority of the respondents had a range of monthly income from MYR 500 to 2000 (45.3%), and the mean income was MYR 2409.80 ± 2325.85. The majority of the respondents in this study also had a long period of survivorship, in which 61.7% of them had survived for more than five years after they had been diagnosed.

As three dietary factors (response variables), i.e., dietary energy density, saturated fat and dietary fiber were included in the RRR analysis, three dietary patterns were identified based on the combined dietary factors. The characteristics of the three dietary patterns are displayed in Table 2. The first dietary pattern presented with the maximum percent of variation explained in all response variables, 34.6%, compared to only 16.3% and 9.1% for dietary patterns 2 and 3, respectively. In addition, the first dietary pattern, which was positively correlated with DED (energy-dense; r = 0.67), SFA (high-SFA; r = 0.36) but negatively correlated with DF (low-fiber; r = −0.65), was shown to be the most pragmatic dietary pattern to be interpreted and in line with the hypothesized link with breast cancer risk. Dietary patterns that explain more than 20% of variation in all response variables were usually retained for further analysis [40]. The other two dietary patterns in the current study were not easily interpretable and are not hypothesized to be associated with the risk of breast cancer risk and mortality. Hence, only the first dietary pattern, the ‘energy-dense, high-SFA and low-fiber’ DP was highlighted for further analysis in this study.

**Figure 1:**
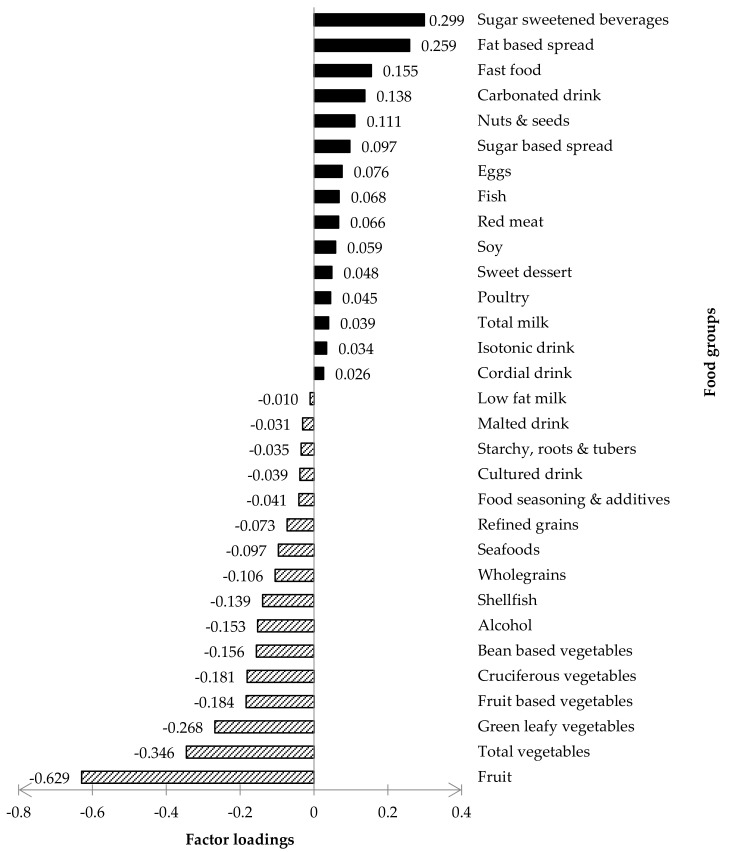
Factor loadings for the ‘energy-dense, High-SFA and Low-Fiber’ DP.

Figure 1 presented the factor loading of the ‘energy-dense, high-SFA and low-fiber’ DP. Intake of foods with a positive factor loading increased the DP z-score, whilst the intake of foods with a negative factor loading decreased the DP z-score. According to previous studies by Jacobs et al. [41] and Kim, Shin, and Song [42], food groups with factor loading ≥0.20 and ≤−0.20 were significant and considered as the largest positive or negative contribution to the dietary pattern z-scores, respectively. In this present study, the ‘energy-dense, high-SFA, low-fiber’ DP was strongly characterized by sugar-sweetened beverages and fat-based spreads (≥0.20 factors loadings) but negatively characterized by fruits, total vegetables, and green vegetables (≤−0.20 factors loadings). Therefore, these five food groups were considered as key foods and were further investigated with the biomarker of interest (HMW adiponectin and leptin) in this present study. The factor loadings for the other two neglected DPs are presented as Supplementary Materials (Figures S1 and S2).

Table 3 summarizes the multiple linear regression analysis of the relationship between the identified DP, key five food groups (those with high factor loadings) and adipokines (HMW adiponectin and leptin) concentration. Only HMW adiponectin had a significant inverse relation with the ‘energy-dense, high-SFA, low-fiber’ DP (β = −0.410; 95% CI = −0.806, −0.014; *p* = 0.043), but no relationship was observed with leptin, independent of age, BMI, cancer stage, duration since diagnosis, education level and occupation status. The findings show that for every 1-unit increase in the ‘energy-dense, high-SFA, low-fiber’ DP z-score, there is a reduction by 0.41 μg/mL in HMW adiponectin. Meanwhile, no significant findings were observed for the other two rejected DP (Supplementary Materials Table S1).

In addition, regression analysis between the key food groups with adipokines concentration showed no significant relationship with HMW adiponectin, but for leptin, green leafy vegetables showed a negative association with leptin concentration even after adjusting for confounding variables (β = −0.079; 95% CI = −0.151, −0.007; *p* = 0.032). This could be interpreted as for every 1 g per day increase in the green leafy vegetable intake, there is a reduction by 0.079 ng/mL in leptin.

## 4. Discussion

The present study identified three dietary factors, i.e., DED, SFA and DF, which were hypothesized to be associated with breast cancer survival and mortality. This was the first study to date that utilized DED, SFA and DF as response variables in RRR analysis for breast cancer survival outcomes. However, an ‘energy-dense, high-SFA and low-fiber’ DP in this present study seemed to be similar to the dietary pattern characterized in another study among breast cancer survivors which was named as ‘Western’ DP [43]. This Western DP showed high factor loading for dessert, high-fat dairy, processed and red meat, whereas low factor loadings were observed for fruit, vegetables, and whole grain. Nonetheless, similar characteristics of DP were found by Vrieling et al. [32]; however, the DP was named as an ‘unhealthy’ DP with a high factor loading of red meat, processed meat, deep-frying, and low factor loading for fruits and vegetables. These previous studies concluded that lower intake of the ‘Western’ DP may protect against mortality from causes unrelated to breast cancer and an increasing intake of an ‘unhealthy’ dietary pattern may increase the risk of non-breast cancer mortality.

The ‘energy-dense, high-SFA and low-fiber’ DP in this study was significantly and inversely related to HMW adiponectin concentration after adjusting for the potential confounding factors. This inverse relationship appeared to be consistent with other research which also found a negative relationship between the Traditional English pattern [20], the “Izakaya” pattern [21] and the Western pattern [44,45] with adiponectin concentration. As compared to DP in this current study, all dietary patterns observed earlier shared similar characteristics of high consumption levels of energy-dense food such as fried foods, fast foods, processed meat, sugar, refined grains intake and have low consumptions of vegetables, fruits, wholegrain, and low-fat dairies.

This significant negative relationship between unhealthy DPs and adiponectin concentration might be explained by the role of adiponectin in regulating food intake and energy expenditure [46]. In terms of energy metabolism, adiponectin acts as a starvation hormone that enhances energy storage by stimulating food intake and suppressing energy expenditure. Therefore, a decline in HMW adiponectin might explain the fact that the energy storage in the body has exceeded and preceded the development of insulin resistance. Previous studies have found positive relationships between dietary patterns that were characterized by healthy food consumption and adiponectin concentration [8]. This further supported the explanation of the effects of dietary pattern towards circulating adiponectin [20,21,22,45]. Furthermore, breast cancer patients showed that a reduction in the concentration of HMW adiponectin had an important effect on insulin resistance and metabolic syndrome, and this was associated with an increased risk of breast cancer mortality [47]. Hence, it could conceivably be suggested that a healthy DP has benefits in improving adiponectin concentration, while an unhealthy DP lowers the serum adiponectin levels.

Nonetheless, no significant relationship was found between leptin concentration and the ‘energy-dense, high-SFA and low-fiber’ DP among breast cancer survivors in this present study. This result was similar to earlier studies’ observations, in which leptin concentration was not independently associated with the ‘Western’ DP (unhealthy DP characterized by red and processed meats, high-energy drinks, refined grains, pizza/lasagna, eggs, fats, and snacks/sweets) [45,48]. In contrast, several previous studies reported a positive association between the ‘Western’ DP with serum leptin concentration [19,49]. Different sample characteristics from different studies, in terms of sex, age, ethnicity, culture, food habits and potential confounding factors, may show different outcomes.

Although our study did not show a significant association between serum concentration of leptin and DP, it did provide evidence that circulating leptin had a significant negative relationship with green leafy vegetables. This finding mirrored those of the previous studies that have examined reductions in circulating leptin levels following healthy hypocaloric diets and regular physical activity [50,51]. Leptin sensitivity was increased with a high amount of fiber intake and has led to control in the secretion of leptin [52]. According to the studies by Harris et al. [53] and Khan et al. [15], a decline in leptin concentration was directly associated with reduced breast cancer recurrence and mortality. Thus, to improve survival and prevent the recurrence of breast cancer, a healthy diet particularly diets high in vegetables is recommended. This coincides with the central recommendation by WCRF/AICR to “Eat mostly food of plant origin, with a variety of non-starchy vegetables and of fruit every day with unprocessed cereals and/or pulses within every meal” [30,54]. It is also known that leptin-induced mammalian target of rapamycin (mTOR) activation may have implications for obesity-related pathophysiological conditions such as breast cancer [55]. Whether the findings from the current study depend on the altered mTOR pathway warrants further investigation.

Overall, this study looks into the DPs practiced among a group of breast cancer survivors in East Coast of Peninsular Malaysia by using RRR analysis, a hybrid method that had used a priori information to identify a nutrient-specific DP. Additionally, this study had contributed significant evidence concerning the health status of breast cancer survivor in Malaysia. However, the present study should be interpreted with caution due to the established limitations attributed to the cross-sectional study design. The current study also did not assess the physical activity level of breast cancer survivors which is one of the important factors that may influence adipokines concentration [14]. Therefore, there is a need to perform a prospective study to obtain stronger evidence that the converse of the identified DP does indeed predictably improve breast cancer survivorship.

## 5. Conclusions

HMW adiponectin concentration among breast cancer survivors in the East Coast of Peninsular Malaysia negatively associated with ‘energy-dense, high-SFA and low-fiber’ DP, which was characterized by the high level of consumption of sugar-sweetened drinks and fat-based spreads but low consumption levels of fruits, total vegetables, and green leafy vegetables. The present finding and those of some previous studies support the hypothesis that an ‘energy-dense, high-SFA and low-fiber’ DP or a similar unhealthy DP is associated with lower beneficial adipokines concentration. The high prevalence of both obesity and overweight might be fundamental to the alternating HMW adiponectin and leptin concentrations among respondents in this study. Future work should consider the long-term effects of adopting healthy dietary practices on breast cancer recurrence and other disease risks in this group of women as an important part of survival after cancer.

## Figures and Tables

**Table 1:** Characteristics of breast cancer survivors.

	n (%)	Mean ± SD	Range
Age		52.7 ± 7.9	37–72
Ethnic			
Malay	121 (94.5)		
Chinese	7 (5.5)		
Marital Status			
Single	5 (3.9)		
Married	99 (77.3)		
Widowed	20 (15.6)		
Divorced	4 (3.1)		
Education level			
None	1 (0.8)		
Primary	11 (8.6)		
Secondary	76 (59.4)		
College/University	40 (31.2)		
Occupational Status			
Working	66 (51.6)		
Not Working	62 (48.4)		
Monthly income (MYR)		2409.80 ± 2325.85	100–12,000
≤500	22 (17.2)		
500–2000	58 (45.3)		
≥2000	48 (37.5)		
Duration since diagnosis (years)		7.14 ± 3.92	2–33
≤5 year	49 (38.3)		
>5 year	79 (61.7)		
Cancer stage			
Stage I	23 (18.0)		
Stage II	71 (55.5)		
Stage III	34 (26.5)		
Body Weight (kg)		66.48 ± 12.52	38–115
Body mass index (kg/m^2^)		27.72 ± 5.03	15–50
Underweight	3 (2.3)		
Normal	29 (22.7)		
Overweight	58 (45.3)		
Obese	38 (29.7)		
Waist circumference (cm)		87.98 ± 11.30	56–125
≤ 80 cm	28 (21.9)		
> 80 cm	100 (78.1)		
HMW Adiponectin (μg/mL) ^a^		3.69 ± 2.65	0.17–14.73
Leptin (ng/mL) ^b^		45.85 ± 19.45	2.29–88.44

^a^ Intra-assay coefficients variation, CV HMW (high-molecular weight) adiponectin = 13.19%; ^b^ Intra-assay coefficients variation, CV Leptin = 9.75%.

**Table 2:** Characteristics of dietary patterns by reduced rank regression.

	Explained Variation (%)					Correlation Coefficient		
	All Food Intakes (Current)	All Responses (Current)	DED (kcal/g)	SFA (%E)	DF (g/kcal)	DED (kcal/g)	SFA (%E)	DF (g/kcal)
DP 1	5.3	34.6	46.7	13.6	43.4	0.67	0.36	−0.65
DP 2	3.5	16.3	52.1	55.7	44.9	−0.33	0.93	0.17
DP 3	4.0	9.1	64.1	55.9	60.0	0.66	0.10	0.74

DP: dietary pattern; DED: dietary energy density; SFA: saturated fatty acid; DF: dietary fibre; %E: percentage of energy intake.

**Table 3:** Relationship between dietary pattern and food groups with adipokines.

	HMW Adiponectin		Leptin	
	β (95% CI)	*p*-Value	β (95% CI)	*p*-Value
**‘Energy dense, High-SFA and low-fiber’ DP**				
^a^ Unadjusted	−0.369 (−0.777,0.039)	0.075	1.701 (−1.350,4.752)	0.271
^b^ Adjusted	−0.410 (−0.806,−0.014)	0.043 *	0.815 (−2.110,3.740)	0.581
**Sugar sweetened beverages (mL/day)**				
^a^ Unadjusted	0.000 (−0.003,0.002)	0.765	−0.012 (−0.031,0.008)	0.235
^b^ Adjusted	0.000 (−0.003,0.002)	0.826	−0.009 (−0.028,0.010)	0.357
**Fat based spread (g/day)**				
^a^ Unadjusted	−0.071 (−0.261,0.119)	0.459	−0.290 (−1.692,1.112)	0.682
^b^ Adjusted	−0.076 (−0.267,0.114)	0.428	−0.286 (−1.650,1.077)	0.677
**Fruits (g/day)**				
^a^ Unadjusted	0.003 (0.000,0.006)	0.050 *	0.002 (−0.019,0.023)	0.861
^b^ Adjusted	0.003 (0.000,0.006)	0.055	0.002 (−0.019,0.023)	0.842
**Total vegetables (g/day)**				
^a^ Unadjusted	−0.004 (−0.014,0.006)	0.419	−0.080 (−0.152,−0.009)	0.027 *
^b^ Adjusted	−0.008 (−0.017,0.002)	0.119	−0.067 (−0.137,0.002)	0.057
**Green leafy vegetables (g/day)**				
^a^ Unadjusted	−0.006 (−0.016,0.004)	0.216	−0.092 (−0.165,−0.018)	0.016 *
^b^ Adjusted	−0.009 (−0.020,0.001)	0.068	−0.079 (−0.151,−0.007)	0.032 *

β Regression coefficient; ^a^ crude regression coefficient by simple linear regression; ^b^ adjusted regression coefficient by multiple linear regression, controlled for energy intake (kcal/d), age (years), BMI (kg/m^2^), cancer stage (stage of cancer upon diagnosed either stage I/II/III), duration since diagnosis (years), education level (primary, secondary, college/university) and occupation status; only food groups with strongest positive factor loadings (≥0.2) and strongest negative factor loadings (≤−0.2) are shown. * statistically significant difference (*p* < 0.05).

## Data Availability

The data presented in this study are available on request from the corresponding author (M.R.S). The data are not publicly available due to the privacy of research participants and ethical restrictions.

## Supplementary Materials

The following are available online at https://www.mdpi.com/article/10.3390/nu13103339/s1, Figure S1: Factor loadings for DP 2, i.e., ‘low energy-density, high-SFA and high-fiber’, Figure S2: Factor loadings for DP 3, i.e., ‘energy-dense, high-SFA and high-fiber’, Table S1: Relationship between dietary patterns (DP 2 and DP 3) and adipokines.
