# Supplementary material for: ‘Energy-Dense, High-SFA and Low-Fiber’ Dietary Pattern Lowered Adiponectin but Not Leptin Concentration of Breast Cancer Survivors
Source: Nutrients. 2021 Sep 24;13(10):3339. doi: 10.3390/nu13103339 (PMC8540181; doi:10.3390/nu13103339)
Supplement: Supplementary file 1 [file nutrients-13-03339-s001.zip › nutrients-1366907-supplementary.pdf]

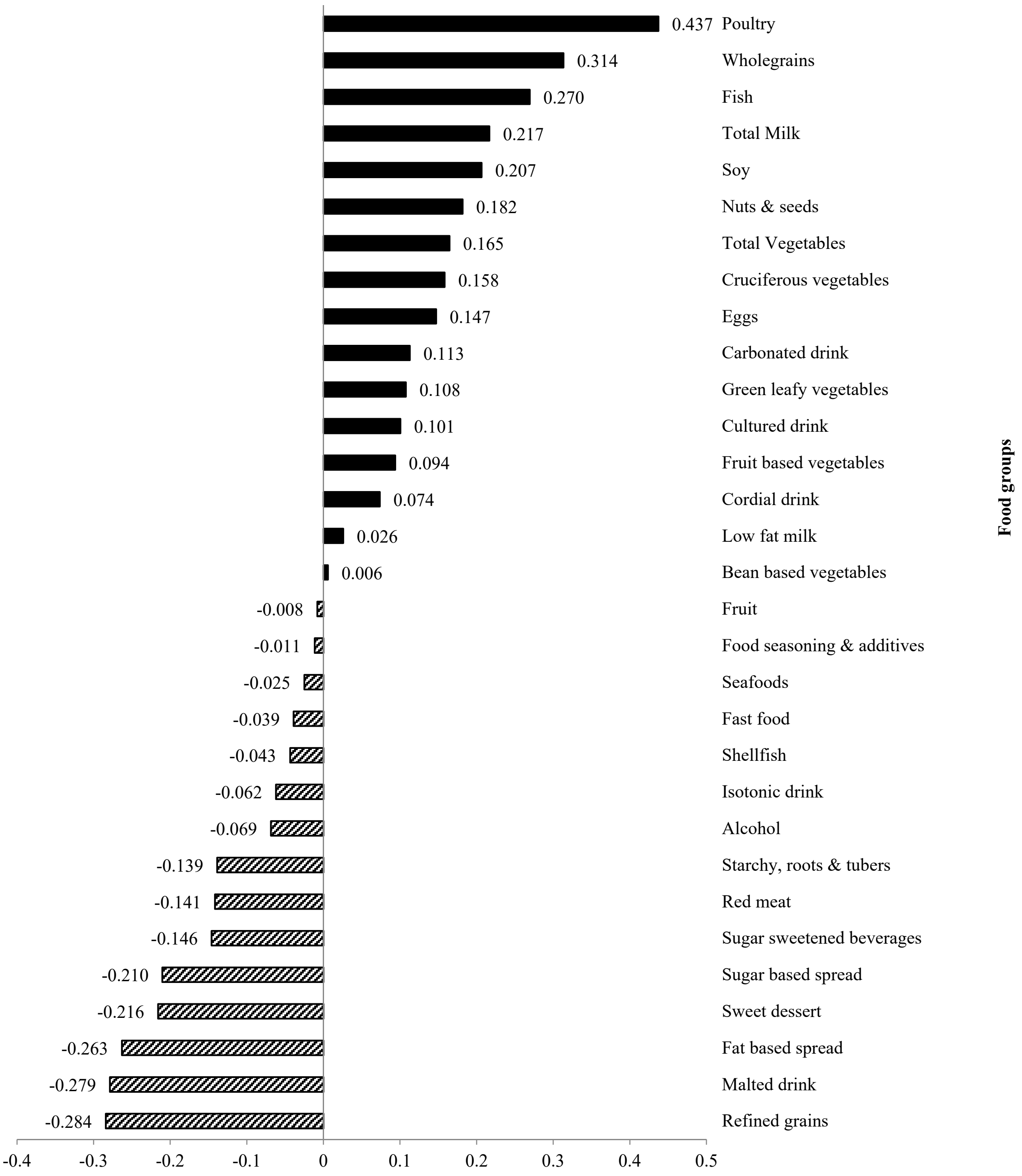

Figure S1. Factor loadings for DP 2 i.e., 'Low Energy-dense, High-SFA and High-Fiber'

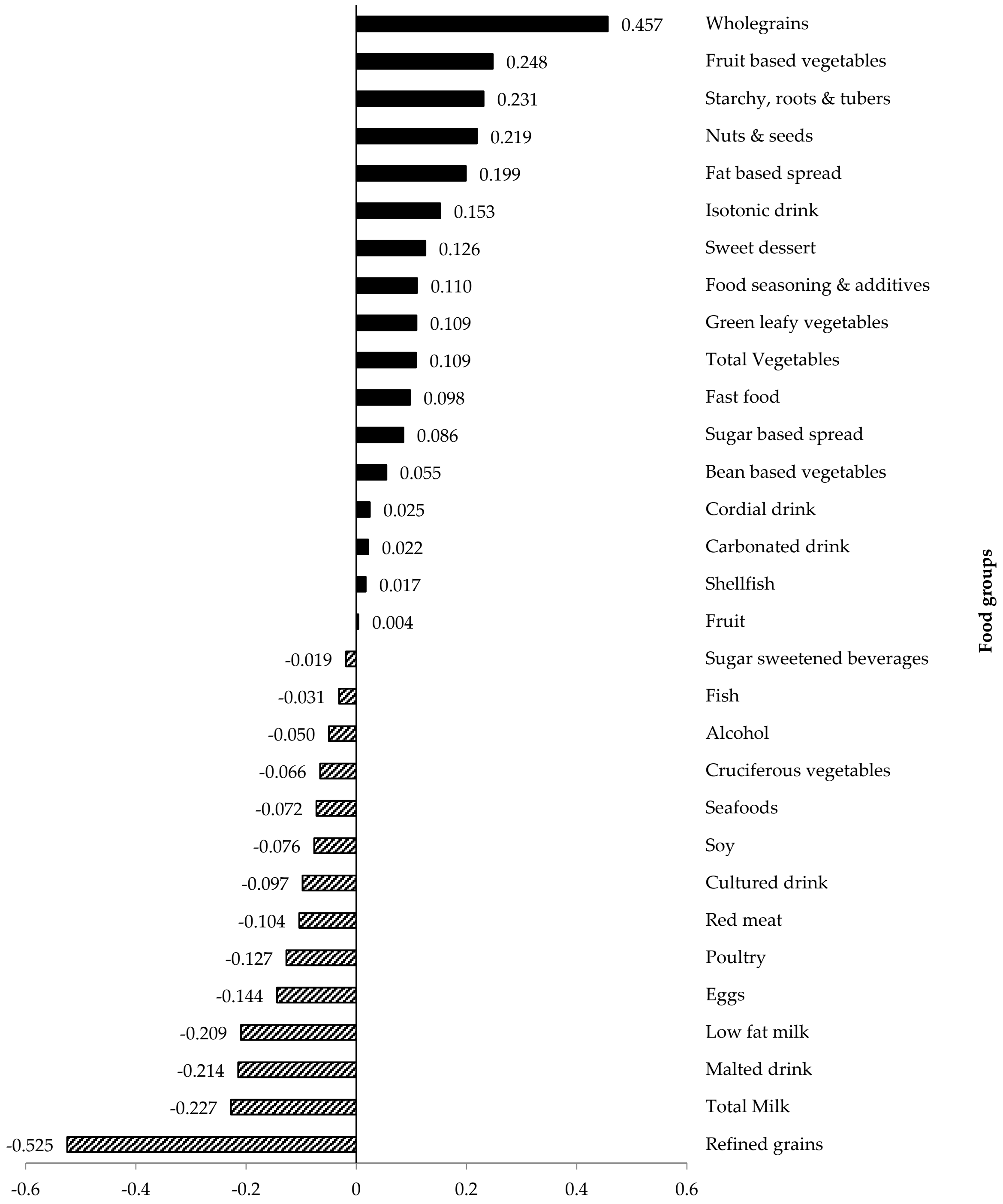

Figure S2. Factor loadings for DP 3 i.e., 'Energy-dense, High-SFA and High-Fiber'

Table S1. Relationship between dietary patterns (DP 2 & DP 3) and adipokines

	HMW Adiponectin		Leptin	
	β (95% CI)	p-value	β (95% CI)	p-value
**DP 2; 'Low Energy-dense, High-SFA and High-Fiber'**				
[a]Unadjusted	0.007 (-0.593,0.606)	0.982	-6.686 (-10.888,2.483)	0.102
[b]Adjusted	-0.287 (-0.880,0.305)	0.338	-4.656 (-8.773,0.539)	0.270
**DP 3; 'Energy-dense, High-SFA and High-Fiber'**				
[a]Unadjusted	0.448 (-0.326,1.222)	0.254	0.789 (-4.997,6.574)	0.787
[b]Adjusted	0.385 (-0.401,1.172)	0.333	3.837 (-1.765,9.440)	0.177

β Regression coefficient; [a] Crude regression coefficient by simple linear regression; [b] Adjusted regression coefficient by multiple linear regression, controlled for energy intake (kcal/d), age (years), BMI (kg/m^2), cancer stage (stage of cancer upon diagnosed either stage I/II/III), duration since diagnosis (years), education level (primary,secondary,college/university) and occupation status.
